# The Medical Inspection of School Children

**Published:** 1907-12-07

**Authors:** 


### The Hospital

A Journal of

The Medical Sciences and Hospital Administration.

New Series. No. 37, Vol. II. [No. 1109, Vol. XLIII.] SATURDAY, DECEMBER 7, 1907.

### The Medical Inspection of School Children.

On January 1, 1908, there will come into operation an Act of Parliament which imposes on every educational authority the duty of securing the medical inspection of children in attendance at public elementary schools. In order that this duty may be effectively discharged, and that suitable and uniform machinery may be adopted, the Board of Education has issued a memorandum to the various local authorities, and the terms of this memorandum closely concern all who are interested in the public health and physical fitness of the rising generation. In addition, there are, in the Board's circular, provisions dealing with the position and duties of medical officers charged with the details of inspection; and it well may be that out of the position now officially defined there may issue other questions which will demand the close attention of the medical profession. We, therefore, suggest to our readers the advisability of giving to the Board's memorandum a somewhat careful study, and we will here briefly indicate the broader outlines on which it is framed.

In the first place it is to be noted that the Board of Education has refused the claim that the inspection of schools and of school children ought to be treated as a separate and special department, and placed under the control of medical officers distinct and peculiar to itself. On the contrary, the memorandum insists that the work is an integral factor in the health of the nation, and that it must be carried out in intimate conjunction with the public health authorities, and under the direct supervision of the medical officer of health. Arguments both on one side and the other have been made familiar to the profession during recent months, and there is no need to repeat them. The decision of the Board of Education has, so far, settled the matter, and it will, we believe, command the sympathy of the great majority of the profession. Especially forcible in this direction are the considerations that, to be successful, inspection must extend over the whole environment of the child, that school hygiene cannot be separated from home hygiene, and that this, in turn, is intimately bound up with the hygienic condition of the community. The result of the decision of the Board of Education on this point will be that existing medical officers of health will be called upon to advise the local educational authorities in regard to the work which is demanded by the new Act, and also to supervise that work when it has been organised. Manifestly the greater part of the new duties will have to be deputed, and, granted that co-ordination is secured, the Board will approve the appointment as school medical officer of either a local medical officer of health, a Poor-law medical officer, a private practitioner, or, as occasion requires, a skilled specialist. In making such appointments preference will be given to practitioners who have had an adequate training in State medicine, or experience in school hygiene, or special opportunities for the study of disease in children.

The character and degree of medical inspection to be practised are in harmony with the view already defined that school hygiene is merely a part of the hygienic condition of the community, and that through this much may be done both to check the occurrence and spread of disease, and to raise the standard of physical fitness. Hence it is the "broad, simple necessities of a healthy life," rather than the accumulation of scientific statistics, that are to be kept in mind. Attention is to be paid to the cleansing, ventilation, and lighting of schoolrooms, as well as to the bodies and minds of the children, and the facts noted with regard to each individual child are to be recorded in accordance with an official plan and schedule. The Board of Education has authority under the Act to fix the frequency of medical inspection, and it has decided that not less than three inspections during the school life of the child will be necessary to secure the results desired. The first of these should take place on the child's admission to the school, the second in the third year of school life, and the third either in the sixth year of school life or shortly before the school period is completed. It is hoped, however, that this minimum demand will not prove the limit of the measure of medical inspection. The local authorities are, indeed, to be encouraged to sanction various inquiries bearing upon the health and physical fitness of the children under their charge. Periodical re-testing of the eyesight, an annual measurement of height and weight, careful anthropometric surveys—these and similar investigations are mentioned as "desirable where practicable, and calculated to advance school hygiene."

The work thus outlined is obviously the opportunity for the application of expert medical knowledge and common-sense, not only to the promotion of the well-being of individual children, but also to the education of the public conscience in regard to the value of health and to the relation between health and hygiene. The results which will follow the adequate discharge of such duties must necessarily be of the highest value. Undoubtedly delicate questions will arise, and more particularly in relation to the provision of treatment for children found unable, on grounds of health or physical defect, to profit by their educational opportunities. These will have to be faced in due course, and to their just settlement the medical profession will be willing to contribute an effective share.

